# A Bioinformatics Tool for the Prediction of DNA N6-Methyladenine Modifications Based on Feature Fusion and Optimization Protocol

**DOI:** 10.3389/fbioe.2020.00502

**Published:** 2020-06-04

**Authors:** Jianhua Cai, Donghua Wang, Riqing Chen, Yuzhen Niu, Xiucai Ye, Ran Su, Guobao Xiao, Leyi Wei

**Affiliations:** ^1^Fujian Provincial Key Laboratory of Information Processing and Intelligent Control, College of Computer and Control Engineering, Minjiang University, Fuzhou, China; ^2^College of Mathematics and Computer Science, Fuzhou University, Fuzhou, China; ^3^Department of General Surgery, Heilongjiang Province Land Reclamation Headquarters General Hospital, Harbin, China; ^4^College of Computer and Information Sciences, Fujian Agriculture and Forestry University, Fuzhou, China; ^5^Department of Computer Science, University of Tsukuba, Tsukuba, Japan; ^6^College of Intelligence and Computing, Tianjin University, Tianjin, China; ^7^School of Software, Shandong University, Jinan, China

**Keywords:** DNA N6-methyladenine site, machine learning, feature representation, sequence-based predictor, feature fusion

## Abstract

DNA N^6^-methyladenine (6mA) is closely involved with various biological processes. Identifying the distributions of 6mA modifications in genome-scale is of great significance to in-depth understand the functions. In recent years, various experimental and computational methods have been proposed for this purpose. Unfortunately, existing methods cannot provide accurate and fast 6mA prediction. In this study, we present 6mAPred-FO, a bioinformatics tool that enables researchers to make predictions based on sequences only. To sufficiently capture the characteristics of 6mA sites, we integrate the sequence-order information with nucleotide positional specificity information for feature encoding, and further improve the feature representation capacity by analysis of variance-based feature optimization protocol. The experimental results show that using this feature protocol, we can significantly improve the predictive performance. Via further feature analysis, we found that the sequence-order information and positional specificity information are complementary to each other, contributing to the performance improvement. On the other hand, the improvement is also due to the use of the feature optimization protocol, which is capable of effectively capturing the most informative features from the original feature space. Moreover, benchmarking comparison results demonstrate that our 6mAPred-FO outperforms several existing predictors. Finally, we establish a web-server that implements the proposed method for convenience of researchers' use, which is currently available at http://server.malab.cn/6mAPred-FO.

## Keypoints

- In this study, we present 6mAPred-FO, a powerful bioinformatics tool for the prediction of 6mA sites.
- In 6mAPred-FO, we integrate the sequence-order information with nucleotide positional specificity information for feature encoding, and further improve the feature representation capacity by feature optimization.
- Comparative results showed that the proposed 6mAPred-FO significantly outperforms several existing predictors.
- We have established a webserver implementing the proposed 6mAPred-FO. It is publicly accessible at http://server.malab.cn/6mAPred-FO.

## Introduction

N^6^-methyladenine (6mA), as a dynamic DNA epigenetic modification, has been extensively discovered in the following three species: bacteria, archaea and eukaryotes (O'Brown and Greer, 2016). The newly studies have indicated that 6mA modification participates in a wide spectrum of important biological processes. In prokaryotes, for example, 6mA has been found to be closely correlated with a series of DNA activities, such as replication (Campbell and Kleckner, 1990; Li et al., 2019), repair (Pukkila et al., 1983), transcription (Robbins-Manke et al., 2005), and cellular defense (Luria and Human, 1952; Linn and Arber, 1968; Meselson and Yuan, 1968). In addition, some studies have demonstrated that 6mA can act as an epigenetic mark in Phytophthora genomes and there may be a relationship between patterns of 6mA methylation and adaptive evolution in these important plant pathogens (Chen H. et al., 2018). Besides, recent study demonstrated that DNA 6mA modification plays a significant role in cell fate transition of mammalian cells as well (Liang et al., 2016; Liao et al., 2016). Therefore, it is very indispensable to determine the distribution of 6mA modification sites in genome-scale to systematically interpret its biological functions.

To solve this problem, experimental efforts have been proposed, such as ultra-high performance liquid chromatography coupled with mass spectrometry (UHPLC-ms/ms) (Greer et al., 2015), capillary electrophoresis and laser-induced fluorescence (CE-LIF) (Krais et al., 2010), methylated DNA immunoprecipitation sequencing (MeDIP-seq) (Pomraning et al., 2009), and single-molecule real-time sequencing (SMRT-seq) (Flusberg et al., 2010). Notably, using mass spectrometry together with SMRT-seq, Zhou et al. obtained the first 6mA profile in rice genome (Zhou et al., 2018). Currently, there is a publicly available database namely “MethSMRT” that integrates multiple 6mA datasets derived from SMRT-seq (Ye et al., 2017). Although considerable progress has been made, the use of the high-throughput sequencing techniques is very limited as it is laborious and expensive.

Recently, as the rapid increase of the experimentally validated 6mA sites, more research efforts have been focused on the development of data-driven computational methods, especially machine learning based prediction methods. For instance, Chen et al., proposed the first machine learning based 6mA site predictor, named “i6mA-Pred,” to predict 6mA sites in rice genome (Chen et al., 2019). The i6mA-Pred used nucleotide chemical properties and nucleotide frequency as features to formulate DNA sequences (Chen et al., 2017) and utilized support vector machine (SVM) to train the predictive model (Chen et al., 2019). The i6mA-Pred model achieved 83.13% in terms of the overall accuracy for identifying 6mA sites (Chen et al., 2019). More recently, researchers have proposed to use deep learning to identify 6mA sites, like iDNA6mA (5-step rule) (Tahir et al., 2019). This model can automatically extract features from DNA sequences by convolution neural network (CNN). Although these models have been proven to be effective and efficient in identifying DNA 6mA sites, the accuracy was not high enough to perform the genome-wide prediction.

In this study, we propose a new bioinformatics predictor, namely “6mAPred-FO.” In this predictor, we aim to capture the discriminative characteristics of 6mA sites by different-view information integration and optimization. Based on the sequential features we extracted, we trained an SVM-based prediction model. Benchmarking comparative results have shown that under the 10-fold cross-validation, our model improves the exiting performance to 87.44% in the overall accuracy. Via further experimental analysis, we found that our performance improvement contributes mainly to our feature integration and optimization strategy. In particular, the nucleotide positional specificity information is complementary to sequence-order information to effectively distinguish 6mA sites from non-6mA sites. We anticipate this tool can be useful to discover new 6mA sites in other species, at least complementary to the high-throughput techniques.

## Materials and Methods

### Benchmark Dataset

A high-quality benchmark dataset is essential for building an effective and unbiased supervised learning model. In this study, we used the same stringent benchmark dataset, which is originally proposed in Chen's study (Chen et al., 2019). In the dataset, the positive samples (sequences with 6mA sites) were obtained from NCBI Gene Expression Omnibus and the single-molecule real-time sequencing (Zhou et al., 2018). Afterwards, they separated out the sites with a modification score of <30 according to the Methylome Analysis Technical Note, and used the CD-HIT (Fu et al., 2012) software to eliminate sequences with the similarity of more than 60% (Chen et al., 2019). The negative samples (sequences without 6mA sites) were obtained from sub-sequences containing GAGG motifs in coding sequences (CDSs) of the rice genome (Zhou et al., 2018). Ultimately, 880 6mA sequences (positive samples) and 880 non-6mA sequences (negative samples) were retained in the dataset.

### Framework of the Proposed 6mAPred-FO

**Figure 1:**
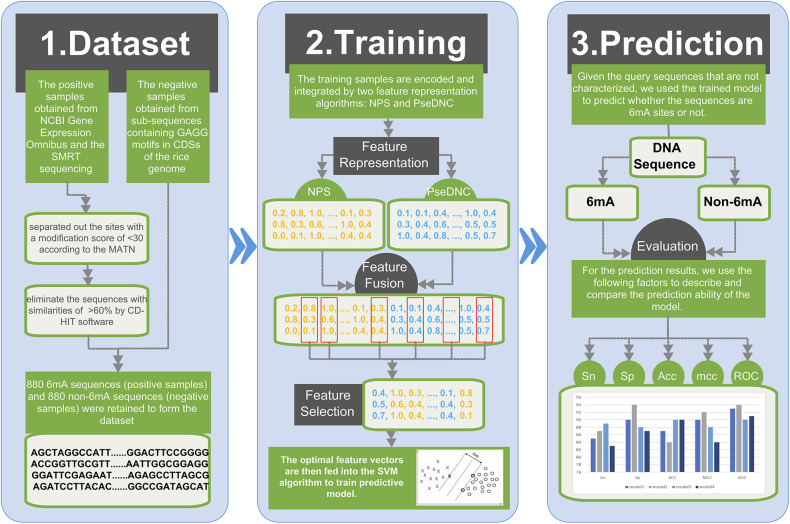
Framework of the proposed 6mAPred-FO. The overall framework is divided into two phases: (1) model training phase and (2) prediction phase. In the model training phase, each DNA sequence is fed to two feature representation algorithms NPS and PseDNC to generate the corresponding features. Afterwards, through the feature fusion and optimization protocol, we yielded the optimal features for each sequence sample. To this end, we train an SVM model with the resulting feature set. In the prediction phase, the model predicts whether the sequence in the test dataset contains 6mA site. And then, compare our model with previous models in terms of ACC, etc. Finally, build a user-friendly web server to provide convenient 6mA site identification and prediction.

Figure 1 illustrates the overall framework of the 6mAPred-FO method for DNA 6mA site prediction. The predictive procedure can be concluded as two phases: model training and prediction. In the training phase, the training samples are encoded and integrated by two feature representation algorithms: NPS (Nucleotide Positional Specificity) and PseDNC (Pseudo Dinucleotide Composition). Afterwards, the features are optimized to obtain the best feature subset for the training set. The resulting feature vectors are then fed into the SVM algorithm to train predictive model. In prediction phase, given the query sequences that are not characterized, we followed the similar procedure to encode the sequences, and used the trained model to predict whether the query sequences are 6mA sites or not.

### Feature Representation Algorithms

To convert DNA sequences into feature vectors that machine learning methods can handle, two feature representation algorithms, Nucleotide Positional Specificity (NPS) and Pseudo Dinucleotide Composition (PseDNC), are introduced for feature representation. Here is a brief introduction to the two algorithms.

#### Nucleotide Positional Specificity (NPS)

In this algorithm, two feature representation descriptors are used to encode the sequences.

The first feature is the positional binary encoding of flanking nucleotide sequence. We adopt the traditional method of flanking window to represent the 6mA site. On the premise that the minimum length 41 can perform well, if the 6mA site is located at both ends of the sequence, we fill the end of the sequence with the gap character “N.” Therefore, in the orthogonal binary coding scheme, we transform nucleotide sequences into numeric vectors by the following rules: the codes of “A (adenine),” “T (thymine),” “C (cytosine),” “G (guanine)” and “N” are “(0, 0, 0, 1),” “(0, 0, 1, 0),” “(0, 1, 0, 0),” “(1, 0, 0, 0),” and “(0, 0, 0, 0),” respectively.

The second feature descriptor of NPS was the position-independent k-mer frequency. We calculated the frequencies of all possible k-mer nucleotides in a site-centered nearby flanking window. However, the vector dimension increases rapidly with the increase of k value, which leads to over-fitting. Thus, we set k to 2, 3, and 4. Finally, the 41-length DNA sequence is transformed into a 500-dimensional vector. More details about this method are available in the Xiang et al. (2016).

#### Pseudo Dinucleotide Composition (PseDNC)

PseDNC combines local and global pattern information of sequences. We use a vector to represent the DNA sequence as given below,

$$\begin{array}{l} R={\left[d_{1}\;{\;d}_{2}\;\cdots \;d_{16}\;d_{16+1}\;\cdots \;d_{16+\lambda }\right]}^{T} \end{array}$$

where

$$d_{u}=\{\begin{array}{c} \frac{f_{u}}{\sum_{i=1}^{16}f_{i}+w\sum_{j=1}^{\lambda }\theta_{j}}\;\left(1\leq u\leq 16\right)\\ \frac{w\theta_{u-16}}{\sum_{i=1}^{16}f_{i}+w\sum_{j=1}^{\lambda }\theta_{j}}\;(16<u\leq 16+\lambda ) \end{array}$$

In the formulation above, *f*_*u*_(*u* = 1, 2, ⋯ , 16) is the normalized occurrence frequency of the u-th non-overlapping dinucleotides in the sequence. The *w* is the weight factor for balancing the component action of pseudo nucleotides. The θ_*j*_ is the *j*-th tier correlation factor that reflects the sequence order correlation between all the *j*-th most contiguous dinucleotides. What's more,

$$\begin{array}{l} \theta_{j}=\frac{1}{L-j-1}\overset{L-j-1}{\underset{i=1}{\sum }}C_{i,\;i+j}\;\left(j=1,2,\cdots \;,\lambda ;\;\lambda <L\right) \end{array}$$

where

$$\begin{array}{l} C_{i,i+j}=\frac{1}{\mu }\overset{\mu }{\underset{g=1}{\sum }}{\left[P_{g}\left(D_{i}\right)-P_{g}\left(D_{i+j}\right)\right]}^{2} \end{array}$$

In the above two formulations, *L* is the length of DNA sequence and the number λ is an integer to reflect the correlation rank which is smaller than *L*. The *C*_*i, i*+*j*_ is correlation function which is given above, where *P*_*g*_(*D*_*i*_) is the numerical value of the *g*-th physicochemical property for the dinucleotide sequence *D*_*i*_ in the DNA, and so as *P*_*g*_(*D*_*i*+*j*_). The μ is the total number of correlation functions counted. It should be noticed that these values of physicochemical property were all subjected into a standard conversion by the formula below before substituting into the *P*_*g*_(*D*_*i*_ ),

$$\begin{array}{l} P_{g}\left(D_{i}\right)=\frac{P_{g}^{0}\left(D_{i}\right)-ave\left(P_{g}^{0}\left(D_{i}\right)\right)}{SD\left\{ave\left(P_{g}^{0}\left(D_{i}\right)\right)\right\}} \end{array}$$

where the symbol *ave*() means getting the average of the values over the 16 different dinucleotides and *SD*{ } means the corresponding standard deviation. In the above equation, $P_{g}^{0}\left(D_{i}\right)$ is the original physicochemical property value for the dinucleotide. In this study, the following three physicochemical properties, namely enthalpy, entropy and free energy, are used to calculate the global or long-range sequence-order effects of the DNA. And their original values are given in Table S1 of Supplementary material.

Ultimately, using this feature descriptor, we obtained 22 features. More details about these formulas can be found in the references Chen et al. (2014, 2015a,b); Liu (2019); Liu et al. (2019b).

### Feature Fusion and Optimization Protocol

Feature fusion has been successfully applied into bio-sequence analysis (Zhang et al., 2017; Tang et al., 2018; Wei et al., 2018a,b; Liu et al., 2019d) and other bioinformatics tasks (Liang et al., 2018; Zhang et al., 2018, 2019a,b; Gong et al., 2019; Wang et al., 2019). It refers to merge different types of feature representations to more comprehensively capture the characteristics of samples from different perspectives. In this study, to make better use of different information, we fused the following two feature representations. One is 500-dimensional feature vector via NPS and the other is 22-dimensional feature vector via PseDNC. Accordingly, we yielded 522-dimensional features.

Generally, the fused feature space probably contains irrelevant or mutual information, impacting the predictive performance. Therefore, feature optimization is a necessary step forwards capturing the most discriminative features from the original feature space, building the optimal predictive model. It can help to eliminate irrelevant or redundant features, so as to reduce feature dimension, improve model accuracy as well as reduce computational cost. On the other hand, selecting relevant features can simplify the model and make it easier to understand the process of data generation. So far, in order to solve these problems, various effective feature optimization methods have been proposed, such as analysis of variance (Feng et al., 2019), binomial distribution (Su et al., 2018), minimal redundancy maximal relevance (Peng et al., 2005), and maximum relevance maximum distance (MRMD) (Zou et al., 2016; Chen W. et al., 2018).

To improve the feature representation ability, we used variance analysis in the filter method for feature selection. Its main idea is to calculate the variance of each feature by function f_classif in sklearn package. By doing so, we obtained the predictive contribution of each feature according to the corresponding f-value. The higher the f-value, the stronger the prediction ability. Afterwards, we selected the features one by one from high to low according to their f-values, and trained the SVM model for each feature subset. Different feature subsets of different dimensions can produce different models, and thus different prediction results can be obtained. The feature subset with the highest accuracy is yielded as the optimal feature subset. The analysis of feature optimization results is discussed in section “RESULTS AND DISCUSSION”.

### Support Vector Machine (SVM)

SVM is a powerful machine learning method for classification, regression and other machine learning tasks. It has been successfully applied in various fields to deal with a series of supervised learning problems (Zhang et al., 2016; Bu et al., 2018; Liu and Li, 2019; Manavalan et al., 2019a,b). The main principle of SVM is to transform the import data into high-dimensional feature space, and then determine the most suitable hyperplane for separating the samples in one class from another. After that, the trained hyperplane can be used to predict the unknown data. Based on this idea, a package namely LibSVM (Chih-chung and Chih-jen, 2011) was established to make the SVM more convenient to use. In this study, we implemented the SVM algorithm by using the LibSVM package. We chose the radial basis kernel (RBF) as a learning function, and optimized the parameters like cost and gamma by grid search to determine the optimal classification hyperplane of SVM. Given a sequence sample, the SVM model can calculate its probability score to be true 6mA sequence. If the probability is more than 50%, it is considered to be the 6mA sequence; otherwise, it is not the 6mA sequence.

### Assessment of Predictive Ability

There are three cross-validation methods namely independent dataset test, n-fold cross-validation test and jackknife test in statistical prediction to evaluate expected success rate of predictors (Manavalan and Lee, 2017; Wei et al., 2017a,d; He et al., 2018; Manavalan et al., 2018; Liu and Zhu, 2019; Liu et al., 2019a). In this study, we used n-fold cross-validation to examine the quality of the model. In the n-fold cross-validation, the dataset was randomly divided into n subsets, of which n-1 subsets were used as training data and the remaining one as testing data. This process would be repeated n times, each time using different testing data in turn. Corresponding accuracy and other evaluation metrics will be obtained in each test, and the average value of the evaluation index obtained from n-time results was used to evaluate the predictor. Generally, multiple n-fold cross-validation (such as 10 times n-fold cross-validation) is needed, and then its mean value is calculated to estimate the accuracy of the predictor.

Four metrics, sensitivity (Sn), specificity (Sp), accuracy (Acc) and Matthew's correlation coefficient (MCC), were used to evaluate the performance of the proposed method. The formulas of these metrics are given below:

$$\{\begin{array}{c} Sn=\frac{TP}{TP+FN}\;0\leq Sn\leq 1\\ Sp=\frac{TN}{TN+FP}\;0\leq Sp\leq 1\\ ACC=\frac{TP+TN}{TP+FP+TN+FN}\;0\leq ACC\leq 1\\ \;MCC=\frac{TP\times TN-FP\times FN}{\sqrt{\left(TN+FN\right)\times \left(TN+FP\right)\times \left(TP+FN\right)\times \left(TP+FP\right)}}\;\\ \;-1\leq MCC\leq 1 \end{array}$$

where, TP (True Positive) represents the number of positive samples correctly predicted; TN (True Negative) represents the number of negative samples correctly predicted; FP (False Positive) represents the number of negative samples incorrectly predicted to be the positives; FN (False Negative) represents the number of positive samples incorrectly predicted to be the negatives.

Moreover, we used the Receiver Operating Characteristic (ROC) curve to measure the overall performance of the predictive model. The area under the ROC curve (AUC) is to quantitively measure the quality of binary classifier. The closer the ROC curve is to the upper left corner, the better the performance of the predictor is. When the AUC value is closer to 0.5, it means that this is a random predictor (Hanley and Mcneil, 1982).

## Results and Discussion

### Comparison of Single and Fused Features

**Figure 2:**
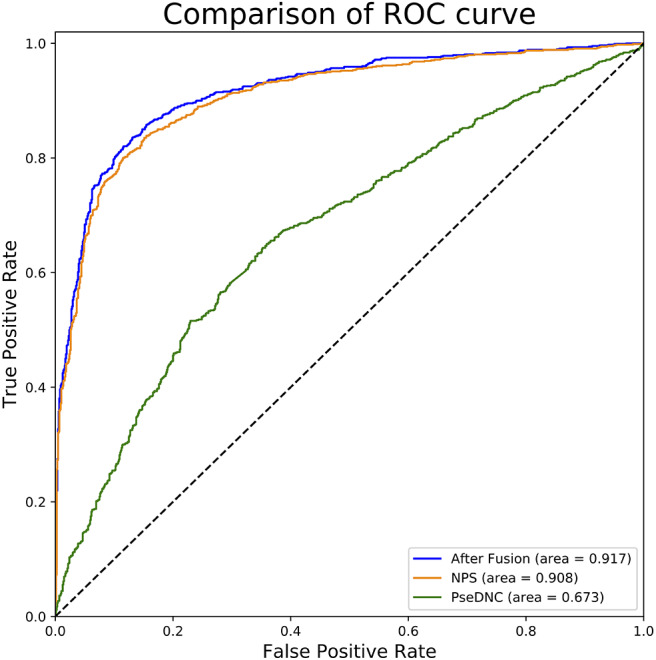
ROC curves of single and fused features. This figure shows the ROC curve results of different features. The green curve characterizes ROC result of PseDNC and the orange one for NPS. The ROC curve of fused features is represented by blue.

In this section, we investigated the impact of the feature fusion protocol on the predictive performance. We compared two feature representations (NPS and PseDNC) with their fusion. They are evaluated with 10-fold cross validation on the same benchmark dataset used in this study. The comparison results are presented in Table 1. It can be seen that the fused features improve the performances in all the metrics. To be specific, the Sn, Sp, ACC, MCC, and AUC is enhanced by 0.34, 1.59, 1, 2, and 0.9%, as compared with the runner-up feature descriptor—NPS. For intuitive comparison, we further compared the ROC curves of different features in Figure 2. Similarly, the fused features show better performance than the single features. From the specific point of view in the Figure 2, the fused feature curve (the blue one) is closer to the upper left corner than the single feature curve. What's more, the AUC value of the fused feature is 0.917, which is higher than that of the single feature. This figure and accurate data can more intuitively support the conclusion above. Together, the results suggest that the information in different features is complementary to better capture the characteristics specificity of 6mA sites.

**Table 1:** Comparison of single feature and fused features.

**Features**	**Sn (%)**	**Sp (%)**	**ACC (%)**	**MCC**	**AUC**
NPS	84.09	83.86	83.98	0.68	0.908
PseDNC	55.91	72.39	64.15	0.29	0.673
Fused Features	84.43	85.45	84.94	0.70	0.917

### Feature Optimization Results

**Figure 3:**
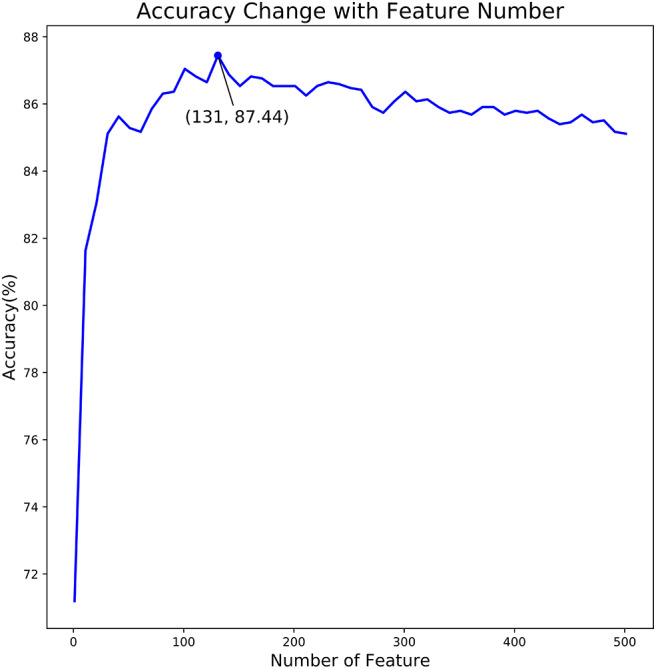
The relationship curve of prediction accuracy and dimension of feature subset. The curve in this figure reflects the change of predictor accuracy with dimension of feature subset.

In the proposed feature optimization strategy, we firstly calculated the classification importance score of each feature in the feature set, and then the features are sorted from high to low according to their scores. Secondly, the feature in the sorted feature set is added to the feature subset one by one. Once a new feature is added to the feature subset, we obtained a new feature subset and train a new SVM model under its default parameters. We evaluated the performance of all feature subsets, respectively. The relationship between prediction accuracy and dimension of feature subset is illustrated in Figure 3.

**Figure 4:**
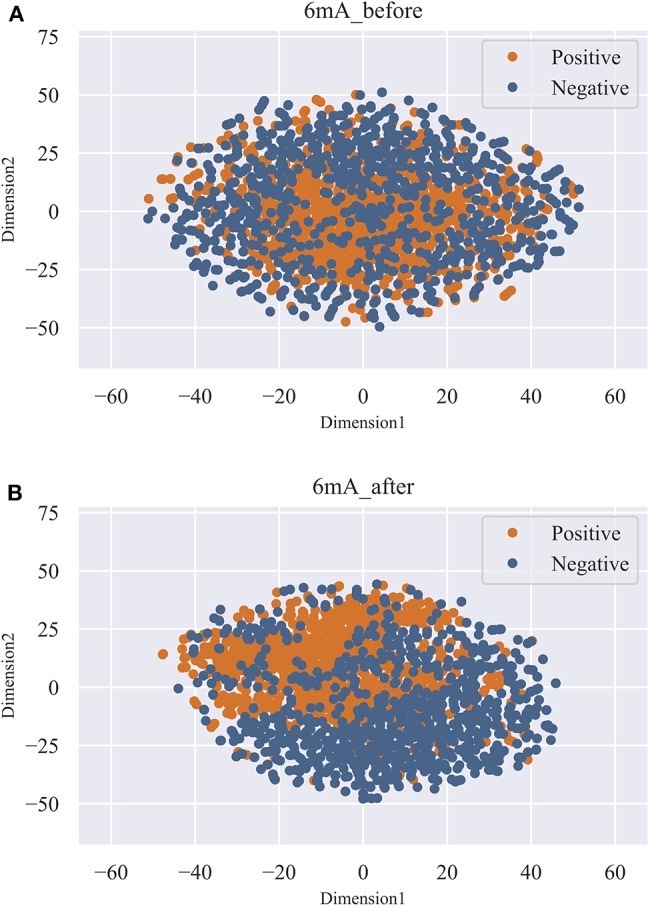
T-SNE visualization of the feature space before and after feature optimization. **(A)** represents the distribution of the positives and negatives in original feature space. **(B)** represents the distribution of the positives and negatives in optimal feature space.

As shown in Figure 3, we observed that the accuracy of the model increased rapidly as the feature number grows. Afterwards, the accuracy slightly declined as the feature number increases. When the feature number reached to 131, the model achieved the highest accuracy of 87.44%. Thus, the 131 features are considered as the optimal and used to train our predictive model. Moreover, the feature optimization results evaluated with other evaluation metrics, like MCC and ROC, can be found in Table S2 of Supporting Information. To visually see how the feature space changes using feature optimization, we further compared the sample distribution between the original feature space and the optimal feature space, as depicted in Figure 4. It can be seen that the positives and negatives in the optimal feature space are more clearly distributed in two clear clusters as the original feature space. It demonstrates that using the feature optimization strategy, it helps to remove the irrelevant features and improve the feature representation ability.

### Comparison of Different Kernel Functions

In this section, we compared the impact of RBF kernel function and other three kernel functions on the performance of our proposed model. They are Linear, Polynomial and Sigmoid. In this study, we used the same dataset to evaluate them. At the same time, they used the best feature subset after our fusion to show the performance. The 10-fold cross validation results can be found in Table S3 of Supplementary material. According to the results in Table S3, we can find easily that SVM model using RBF kernel function achieves the highest prediction accuracy of 87.44% and performs better in other prediction factors. Moreover, with the help of RBF kernel function, AUC of the model is also the highest among several other kernel functions. In general, these results show that RBF kernel function is superior to other kernel functions in this study.

### Comparison With Other Classifiers

**Figure 5:**
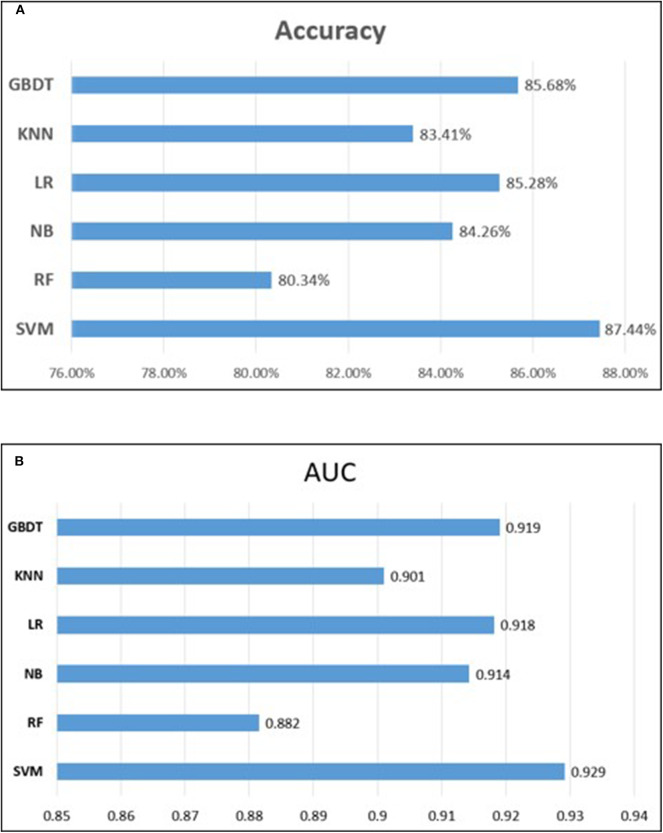
Performance comparison of different classifiers. **(A)** represents the comparison results of prediction accuracy of six classifiers, and **(B)** represents the comparison results of auROC.

**Figure 6:**
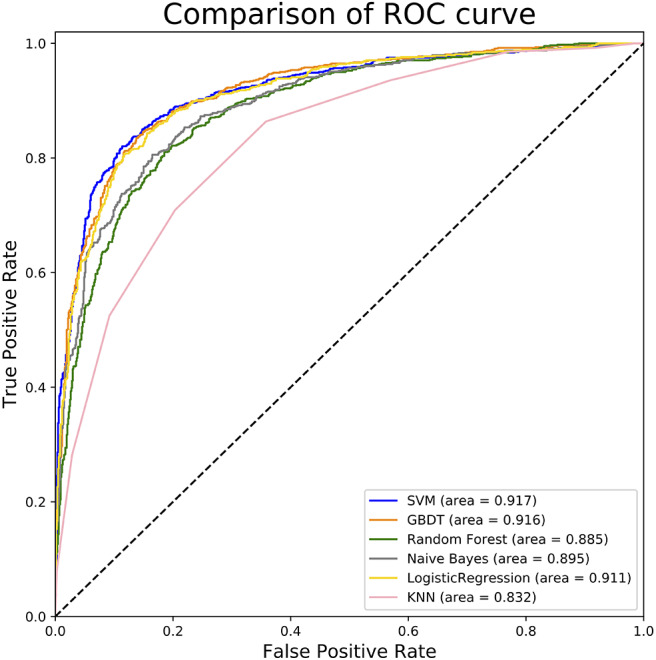
ROC curves of different classifiers. This figure shows the ROC curve results of different classifiers. For example, according to legend, the blue curve characterizes ROC result of SVM and the orange one for GBDT. The value of auROC is also calculated after the name in the legend.

To measure the superiority of SVM, we selected several other classifiers to compare with SVM. There are Gradient Boosting Decision Tree (GBDT) (Liao et al., 2017), K-Nearest Neighbor (KNN), Logistic Regression (LR), Naive Bayes (NB), and Random Forest (RF) (Wei et al., 2017b,c; Lv et al., 2019; Ru et al., 2019). They are evaluated based on the same dataset used in this study with our fused feature set. The 10-flod cross validation results of prediction accuracy and AUC value are illustrated in Figure 5. In Figure 5A represents the comparison results of prediction accuracy of six classifiers, and Figure 5B represents the AUC value. As shown in Figure 5, we observed that the SVM got the highest score among the six classifiers not only in predictive accuracy but also AUC. The 10-fold cross validation results of other evaluation factors are illustrated in Table S4 of Supplementary material, which provide us with more specific classifier performance information. From Table S4, we can see that the SVM also performs better than other classifiers in other performance indicators. For intuitive comparison, we further compared their ROC curves as illustrated in Figure 6. As seen, SVM achieved 0.917 in terms of AUC, which is higher than GBDT and other classifiers. It can be seen from the figure that the ROC curve corresponding to SVM is at the top, which means that SVM has better classification performance than other classifiers. In general, these results demonstrate that SVM is better than other commonly used classifiers in this study.

### Comparison With Existing Predictors

To measure the effectiveness of our predictive model-−6mAPred-FO, we compared the model with i6mA-Pred (Chen et al., 2019) and iDNA6mA (5-step rule) on the same dataset, which are the best two among existing predictors to identify the 6mA site. The results are presented in Table 2. As shown in Table 2, i6mA-Pred obtains the accuracy of 83.13%, sensitivity of 82.95%, specificity of 83.30%, MCC of 0.66 and AUC of 0.886, while our prediction model obtains the accuracy of 87.44%, sensitivity of 86.93%, specificity of 87.95%, MCC of 0.75 and AUC of 0.929. Obviously, our method is superior to i6mA-Pred in all the metrics. Specifically, as compared to i6mA-Pred, our model achieved 4.31%, 3.98%, 4.65%, 0.09 and 0.043 higher in terms of ACC, Sn, Sp, MCC, and AUC, respectively. This demonstrated that our feature representations are more effective to capture the characteristic specificity of 6mA sites. In the Table 2, we also compared our predictor model with iDNA6mA (5-step rule). It can be seen that the accuracy of our 6mAPred-FO is 0.8% higher than iDNA6mA (5-step rule). All the other performance indicators except AUC value are slightly higher than those of iDNA6mA (5-step rule). Generally, it can be concluded that our 6mAPred-FO is better than existing predictors in distinguishing 6mA sites from non-6mA sites.

**Table 2:** Comparison of the proposed 6mAPred-FO with existing predictors.

**Method**	**Sn (%)**	**Sp (%)**	**ACC (%)**	**MCC**	**AUC**
i6mA-Pred	82.95	83.30	83.13	0.66	0.886
iDNA6mA (5-step rule)	86.70	86.59	86.64	0.732	0.931
6mAPred-FO	86.93	87.95	87.44	0.75	0.929

## Conclusions

In this study, we have proposed a new machine learning based 6mA site predictor namely 6mAPred-FO. To sufficiently capture the characteristics of 6mA sites, we have combined the information from two feature representations NPS and PseDNC, and further optimized the features by feature selection. Feature analysis results showed that as compared with the single feature descriptor, the fused features perform better, demonstrating that different information are complementary to improve the predictive performance. Moreover, feature selection is an effective strategy to optimize the feature space and improve the feature representation ability. We have also compared our 6mAPred-FO with existing predictors on benchmark datasets. The comparative results showed that our approach improved the performance significantly in terms of multiple metrics like SN, SP, MCC, and AUC. This suggests that our feature fusion and selection scheme is more effective to represent 6mA sites in comparison with existing features. From our study results, we can make a reasonable inference that the recognition of 6mA site is closely related to the local and global pattern information represented by PseDNC. Then, the position specific information represented by NPS is fused to make our proposed algorithm more accurate for the recognition of 6mA sites. In general, our method provides a more accurate model for biological scientists to identify 6mA site in rice genome. In the future, we will pay more attention on deep learning (Liu et al., 2019c; Zou et al., 2019) for the accuracy improvement.

## Data Availability Statement

The datasets generated for this study can be found in the http://server.malab.cn/6mAPred-FO/Download.html.

## Author Contributions

RC, YN, and DW: conceptualization. XY and RS: data curation. JC: writing—original draft preparation. GX and LW: writing—review and editing. JC: visualization. LW and GX: supervision.

## Conflict of Interest

The authors declare that the research was conducted in the absence of any commercial or financial relationships that could be construed as a potential conflict of interest.
