# Supplementary material for: A Bioinformatics Tool for the Prediction of DNA N6-Methyladenine Modifications Based on Feature Fusion and Optimization Protocol
Source: Front Bioeng Biotechnol. 2020 Jun 4;8:502. doi: 10.3389/fbioe.2020.00502 (PMC7287168; doi:10.3389/fbioe.2020.00502)
Supplement: Supplementary file 1 [file Data_Sheet_1.DOCX]

**Supporting Information**

**Supplementary tables**

**Table S1.** Original values of the three physicochemical properties for the 16 different dinucleotides

| Dinucleotide | Enthalpy  (Ka/mol) | Entropy  (eU) | Free energy  (Ka/mol) |
| --- | --- | --- | --- |
| GG | -12.2 | -29.7 | -3.26 |
| GA | -13.3 | -35.5 | -2.35 |
| GC | -14.2 | -34.9 | -3.42 |
| GT | -10.2 | -26.2 | -2.24 |
| AG | -7.6 | -19.2 | -2.08 |
| AA | -6.6 | -18.4 | -0.93 |
| AC | -10.2 | -26.2 | -2.24 |
| AT | -5.7 | -15.5 | -1.10 |
| CG | -8.0 | -19.4 | -2.36 |
| CA | -10.5 | -27.8 | -2.11 |
| CC | -12.2 | -29.7 | -3.26 |
| CT | -7.6 | -19.2 | -2.08 |
| TG | -7.6 | -19.2 | -2.11 |
| TA | -8.1 | -22.6 | -1.33 |
| TC | -10.2 | -26.2 | -2.35 |
| TT | -6.6 | -18.4 | -0.93 |

**Table S2.** Model performances change with the dimension of feature subset

| Dimension | TP | FN | FP | TN | ACC (%) | SN | SP | MCC | auROC |
| --- | --- | --- | --- | --- | --- | --- | --- | --- | --- |
| 1D | 562 | 318 | 189 | 691 | 71.19 | 0.64 | 0.79 | 0.43 | 0.69 |
| 3D | 689 | 191 | 280 | 600 | 73.24 | 0.78 | 0.68 | 0.47 | 0.73 |
| 5D | 719 | 161 | 240 | 640 | 77.22 | 0.82 | 0.73 | 0.55 | 0.77 |
| 7D | 714 | 166 | 200 | 680 | 79.20 | 0.81 | 0.77 | 0.58 | 0.82 |
| 9D | 701 | 179 | 180 | 700 | 79.60 | 0.80 | 0.80 | 0.59 | 0.83 |
| 11D | 712 | 168 | 155 | 725 | 81.65 | 0.81 | 0.82 | 0.63 | 0.87 |
| 13D | 705 | 175 | 142 | 738 | 81.99 | 0.80 | 0.84 | 0.64 | 0.89 |
| 15D | 719 | 161 | 135 | 745 | 83.18 | 0.82 | 0.85 | 0.66 | 0.90 |
| 17D | 721 | 159 | 143 | 737 | 82.84 | 0.82 | 0.84 | 0.66 | 0.90 |
| 19D | 724 | 156 | 144 | 736 | 82.95 | 0.82 | 0.84 | 0.66 | 0.90 |
| 21D | 720 | 160 | 138 | 742 | 83.07 | 0.82 | 0.84 | 0.66 | 0.90 |
| 23D | 738 | 142 | 137 | 743 | 84.15 | 0.84 | 0.84 | 0.68 | 0.90 |
| 25D | 737 | 143 | 134 | 746 | 84.26 | 0.84 | 0.85 | 0.69 | 0.90 |
| 27D | 732 | 148 | 124 | 756 | 84.55 | 0.83 | 0.86 | 0.69 | 0.92 |
| 29D | 735 | 145 | 125 | 755 | 84.66 | 0.84 | 0.86 | 0.69 | 0.92 |
| 31D | 753 | 127 | 135 | 745 | 85.11 | 0.86 | 0.85 | 0.70 | 0.92 |
| 33D | 744 | 136 | 130 | 750 | 84.89 | 0.85 | 0.85 | 0.70 | 0.92 |
| 35D | 757 | 123 | 127 | 753 | 85.80 | 0.86 | 0.86 | 0.72 | 0.92 |
| 37D | 748 | 132 | 124 | 756 | 85.45 | 0.85 | 0.86 | 0.71 | 0.92 |
| 39D | 749 | 131 | 125 | 755 | 85.45 | 0.85 | 0.86 | 0.71 | 0.92 |
| 41D | 748 | 132 | 121 | 759 | 85.63 | 0.85 | 0.86 | 0.71 | 0.92 |
| 43D | 749 | 131 | 126 | 754 | 85.40 | 0.85 | 0.86 | 0.71 | 0.92 |
| 45D | 738 | 142 | 115 | 765 | 85.40 | 0.84 | 0.87 | 0.71 | 0.92 |
| 47D | 738 | 142 | 120 | 760 | 85.11 | 0.84 | 0.86 | 0.70 | 0.92 |
| 49D | 730 | 150 | 112 | 768 | 85.11 | 0.83 | 0.87 | 0.70 | 0.92 |
| 51D | 738 | 142 | 117 | 763 | 85.28 | 0.84 | 0.87 | 0.71 | 0.92 |
| 53D | 743 | 137 | 125 | 755 | 85.11 | 0.84 | 0.86 | 0.70 | 0.92 |
| 55D | 746 | 134 | 128 | 752 | 85.11 | 0.85 | 0.85 | 0.70 | 0.91 |
| 57D | 742 | 138 | 123 | 757 | 85.17 | 0.84 | 0.86 | 0.70 | 0.92 |
| 59D | 731 | 149 | 114 | 766 | 85.06 | 0.83 | 0.87 | 0.70 | 0.92 |
| 61D | 731 | 149 | 112 | 768 | 85.17 | 0.83 | 0.87 | 0.70 | 0.92 |
| 63D | 729 | 151 | 112 | 768 | 85.06 | 0.83 | 0.87 | 0.70 | 0.92 |
| 65D | 748 | 132 | 119 | 761 | 85.74 | 0.85 | 0.86 | 0.71 | 0.92 |
| 67D | 746 | 134 | 116 | 764 | 85.80 | 0.85 | 0.87 | 0.72 | 0.92 |
| 69D | 745 | 135 | 111 | 769 | 86.02 | 0.85 | 0.87 | 0.72 | 0.93 |
| 71D | 739 | 141 | 108 | 772 | 85.85 | 0.84 | 0.88 | 0.72 | 0.93 |
| 73D | 749 | 131 | 116 | 764 | 85.97 | 0.85 | 0.87 | 0.72 | 0.93 |
| 75D | 747 | 133 | 111 | 769 | 86.14 | 0.85 | 0.87 | 0.72 | 0.93 |
| 77D | 747 | 133 | 109 | 771 | 86.25 | 0.85 | 0.88 | 0.73 | 0.93 |
| 79D | 747 | 133 | 110 | 770 | 86.19 | 0.85 | 0.88 | 0.72 | 0.93 |
| 81D | 747 | 133 | 108 | 772 | 86.31 | 0.85 | 0.88 | 0.73 | 0.93 |
| 83D | 751 | 129 | 109 | 771 | 86.48 | 0.85 | 0.88 | 0.73 | 0.93 |
| 85D | 747 | 133 | 107 | 773 | 86.36 | 0.85 | 0.88 | 0.73 | 0.93 |
| 87D | 751 | 129 | 112 | 768 | 86.31 | 0.85 | 0.87 | 0.73 | 0.93 |
| 89D | 754 | 126 | 111 | 769 | 86.53 | 0.86 | 0.87 | 0.73 | 0.93 |
| 91D | 755 | 125 | 115 | 765 | 86.36 | 0.86 | 0.87 | 0.73 | 0.92 |
| 93D | 756 | 124 | 110 | 770 | 86.70 | 0.86 | 0.88 | 0.73 | 0.93 |
| 95D | 757 | 123 | 111 | 769 | 86.70 | 0.86 | 0.87 | 0.73 | 0.93 |
| 97D | 748 | 132 | 99 | 781 | 86.88 | 0.85 | 0.89 | 0.74 | 0.93 |
| 99D | 750 | 130 | 100 | 780 | 86.93 | 0.85 | 0.89 | 0.74 | 0.93 |
| 101D | 764 | 116 | 112 | 768 | 87.05 | 0.87 | 0.87 | 0.74 | 0.93 |
| 103D | 756 | 124 | 110 | 770 | 86.70 | 0.86 | 0.88 | 0.73 | 0.93 |
| 105D | 758 | 122 | 112 | 768 | 86.70 | 0.86 | 0.87 | 0.73 | 0.93 |
| 107D | 758 | 122 | 111 | 769 | 86.76 | 0.86 | 0.87 | 0.74 | 0.93 |
| 109D | 764 | 116 | 114 | 766 | 86.93 | 0.87 | 0.87 | 0.74 | 0.93 |
| 111D | 764 | 116 | 116 | 764 | 86.82 | 0.87 | 0.87 | 0.74 | 0.93 |
| 113D | 766 | 114 | 115 | 765 | 86.99 | 0.87 | 0.87 | 0.74 | 0.93 |
| 115D | 756 | 124 | 113 | 767 | 86.53 | 0.86 | 0.87 | 0.73 | 0.93 |
| 117D | 754 | 126 | 113 | 767 | 86.42 | 0.86 | 0.87 | 0.73 | 0.93 |
| 119D | 755 | 125 | 112 | 768 | 86.53 | 0.86 | 0.87 | 0.73 | 0.93 |
| 121D | 757 | 123 | 112 | 768 | 86.65 | 0.86 | 0.87 | 0.73 | 0.93 |
| 123D | 758 | 122 | 111 | 769 | 86.76 | 0.86 | 0.87 | 0.74 | 0.93 |
| 125D | 758 | 122 | 109 | 771 | 86.88 | 0.86 | 0.88 | 0.74 | 0.93 |
| 127D | 761 | 119 | 106 | 774 | 87.22 | 0.86 | 0.88 | 0.74 | 0.93 |
| 129D | 763 | 117 | 107 | 773 | 87.27 | 0.87 | 0.88 | 0.75 | 0.93 |
| 131D | 765 | 115 | 106 | 774 | 87.44 | 0.87 | 0.88 | 0.75 | 0.93 |
| 133D | 764 | 116 | 106 | 774 | 87.39 | 0.87 | 0.88 | 0.75 | 0.93 |
| 135D | 760 | 120 | 108 | 772 | 87.05 | 0.86 | 0.88 | 0.74 | 0.93 |
| 137D | 761 | 119 | 108 | 772 | 87.10 | 0.86 | 0.88 | 0.74 | 0.93 |
| 139D | 761 | 119 | 108 | 772 | 87.10 | 0.86 | 0.88 | 0.74 | 0.93 |
| 141D | 759 | 121 | 110 | 770 | 86.88 | 0.86 | 0.88 | 0.74 | 0.93 |
| 143D | 764 | 116 | 113 | 767 | 86.99 | 0.87 | 0.87 | 0.74 | 0.93 |
| 145D | 764 | 116 | 111 | 769 | 87.10 | 0.87 | 0.87 | 0.74 | 0.93 |
| 147D | 763 | 117 | 109 | 771 | 87.16 | 0.87 | 0.88 | 0.74 | 0.93 |
| 149D | 753 | 127 | 107 | 773 | 86.70 | 0.86 | 0.88 | 0.73 | 0.93 |
| 151D | 752 | 128 | 109 | 771 | 86.53 | 0.85 | 0.88 | 0.73 | 0.93 |
| 153D | 756 | 124 | 108 | 772 | 86.82 | 0.86 | 0.88 | 0.74 | 0.93 |
| 155D | 762 | 118 | 110 | 770 | 87.05 | 0.87 | 0.88 | 0.74 | 0.93 |
| 157D | 764 | 116 | 108 | 772 | 87.27 | 0.87 | 0.88 | 0.75 | 0.93 |
| 159D | 761 | 119 | 111 | 769 | 86.93 | 0.86 | 0.87 | 0.74 | 0.93 |
| 161D | 760 | 120 | 112 | 768 | 86.82 | 0.86 | 0.87 | 0.74 | 0.93 |
| 163D | 752 | 128 | 109 | 771 | 86.53 | 0.85 | 0.88 | 0.73 | 0.93 |
| 165D | 751 | 129 | 109 | 771 | 86.48 | 0.85 | 0.88 | 0.73 | 0.93 |
| 167D | 754 | 126 | 107 | 773 | 86.76 | 0.86 | 0.88 | 0.74 | 0.93 |
| 169D | 753 | 127 | 105 | 775 | 86.82 | 0.86 | 0.88 | 0.74 | 0.93 |
| 171D | 753 | 127 | 106 | 774 | 86.76 | 0.86 | 0.88 | 0.74 | 0.93 |
| 173D | 751 | 129 | 106 | 774 | 86.65 | 0.85 | 0.88 | 0.73 | 0.93 |
| 175D | 753 | 127 | 105 | 775 | 86.82 | 0.86 | 0.88 | 0.74 | 0.93 |
| 177D | 753 | 127 | 107 | 773 | 86.70 | 0.86 | 0.88 | 0.73 | 0.93 |
| 179D | 754 | 126 | 109 | 771 | 86.65 | 0.86 | 0.88 | 0.73 | 0.93 |
| 181D | 752 | 128 | 109 | 771 | 86.53 | 0.85 | 0.88 | 0.73 | 0.93 |
| 183D | 752 | 128 | 109 | 771 | 86.53 | 0.85 | 0.88 | 0.73 | 0.93 |
| 185D | 753 | 127 | 110 | 770 | 86.53 | 0.86 | 0.88 | 0.73 | 0.93 |
| 187D | 753 | 127 | 110 | 770 | 86.53 | 0.86 | 0.88 | 0.73 | 0.93 |
| 189D | 752 | 128 | 108 | 772 | 86.59 | 0.85 | 0.88 | 0.73 | 0.93 |
| 191D | 752 | 128 | 109 | 771 | 86.53 | 0.85 | 0.88 | 0.73 | 0.93 |
| 193D | 749 | 131 | 110 | 770 | 86.31 | 0.85 | 0.88 | 0.73 | 0.93 |
| 195D | 750 | 130 | 111 | 769 | 86.31 | 0.85 | 0.87 | 0.73 | 0.93 |
| 197D | 749 | 131 | 108 | 772 | 86.42 | 0.85 | 0.88 | 0.73 | 0.93 |
| 199D | 749 | 131 | 108 | 772 | 86.42 | 0.85 | 0.88 | 0.73 | 0.93 |
| 201D | 749 | 131 | 106 | 774 | 86.53 | 0.85 | 0.88 | 0.73 | 0.93 |
| 203D | 748 | 132 | 108 | 772 | 86.36 | 0.85 | 0.88 | 0.73 | 0.93 |
| 205D | 748 | 132 | 107 | 773 | 86.42 | 0.85 | 0.88 | 0.73 | 0.93 |
| 207D | 746 | 134 | 107 | 773 | 86.31 | 0.85 | 0.88 | 0.73 | 0.93 |
| 209D | 747 | 133 | 110 | 770 | 86.19 | 0.85 | 0.88 | 0.72 | 0.93 |
| 211D | 746 | 134 | 108 | 772 | 86.25 | 0.85 | 0.88 | 0.73 | 0.93 |
| 213D | 756 | 124 | 114 | 766 | 86.48 | 0.86 | 0.87 | 0.73 | 0.93 |
| 215D | 757 | 123 | 114 | 766 | 86.53 | 0.86 | 0.87 | 0.73 | 0.93 |
| 217D | 746 | 134 | 102 | 778 | 86.59 | 0.85 | 0.88 | 0.73 | 0.93 |
| 219D | 758 | 122 | 115 | 765 | 86.53 | 0.86 | 0.87 | 0.73 | 0.93 |
| 221D | 744 | 136 | 101 | 779 | 86.53 | 0.85 | 0.89 | 0.73 | 0.93 |
| 223D | 746 | 134 | 102 | 778 | 86.59 | 0.85 | 0.88 | 0.73 | 0.93 |
| 225D | 754 | 126 | 111 | 769 | 86.53 | 0.86 | 0.87 | 0.73 | 0.93 |
| 227D | 754 | 126 | 109 | 771 | 86.65 | 0.86 | 0.88 | 0.73 | 0.93 |
| 229D | 745 | 135 | 101 | 779 | 86.59 | 0.85 | 0.89 | 0.73 | 0.93 |
| 231D | 745 | 135 | 100 | 780 | 86.65 | 0.85 | 0.89 | 0.73 | 0.93 |
| 233D | 745 | 135 | 99 | 781 | 86.70 | 0.85 | 0.89 | 0.73 | 0.93 |
| 235D | 743 | 137 | 98 | 782 | 86.65 | 0.84 | 0.89 | 0.73 | 0.93 |
| 237D | 741 | 139 | 97 | 783 | 86.59 | 0.84 | 0.89 | 0.73 | 0.93 |
| 239D | 741 | 139 | 99 | 781 | 86.48 | 0.84 | 0.89 | 0.73 | 0.93 |
| 241D | 743 | 137 | 99 | 781 | 86.59 | 0.84 | 0.89 | 0.73 | 0.93 |
| 243D | 740 | 140 | 99 | 781 | 86.42 | 0.84 | 0.89 | 0.73 | 0.93 |
| 245D | 744 | 136 | 101 | 779 | 86.53 | 0.85 | 0.89 | 0.73 | 0.93 |
| 247D | 742 | 138 | 101 | 779 | 86.42 | 0.84 | 0.89 | 0.73 | 0.93 |
| 249D | 742 | 138 | 101 | 779 | 86.42 | 0.84 | 0.89 | 0.73 | 0.93 |
| 251D | 742 | 138 | 100 | 780 | 86.48 | 0.84 | 0.89 | 0.73 | 0.92 |
| 253D | 742 | 138 | 100 | 780 | 86.48 | 0.84 | 0.89 | 0.73 | 0.92 |
| 255D | 737 | 143 | 98 | 782 | 86.31 | 0.84 | 0.89 | 0.73 | 0.92 |
| 257D | 757 | 123 | 117 | 763 | 86.36 | 0.86 | 0.87 | 0.73 | 0.93 |
| 259D | 757 | 123 | 119 | 761 | 86.25 | 0.86 | 0.86 | 0.73 | 0.93 |
| 261D | 742 | 138 | 101 | 779 | 86.42 | 0.84 | 0.89 | 0.73 | 0.92 |
| 263D | 741 | 139 | 100 | 780 | 86.42 | 0.84 | 0.89 | 0.73 | 0.92 |
| 265D | 753 | 127 | 119 | 761 | 86.02 | 0.86 | 0.86 | 0.72 | 0.93 |
| 267D | 753 | 127 | 119 | 761 | 86.02 | 0.86 | 0.86 | 0.72 | 0.93 |
| 269D | 750 | 130 | 119 | 761 | 85.85 | 0.85 | 0.86 | 0.72 | 0.93 |
| 271D | 750 | 130 | 118 | 762 | 85.91 | 0.85 | 0.87 | 0.72 | 0.92 |
| 273D | 751 | 129 | 120 | 760 | 85.85 | 0.85 | 0.86 | 0.72 | 0.93 |
| 275D | 751 | 129 | 120 | 760 | 85.85 | 0.85 | 0.86 | 0.72 | 0.93 |
| 277D | 751 | 129 | 120 | 760 | 85.85 | 0.85 | 0.86 | 0.72 | 0.93 |
| 279D | 749 | 131 | 120 | 760 | 85.74 | 0.85 | 0.86 | 0.71 | 0.92 |
| 281D | 749 | 131 | 120 | 760 | 85.74 | 0.85 | 0.86 | 0.71 | 0.92 |
| 283D | 752 | 128 | 120 | 760 | 85.91 | 0.85 | 0.86 | 0.72 | 0.92 |
| 285D | 750 | 130 | 118 | 762 | 85.91 | 0.85 | 0.87 | 0.72 | 0.92 |
| 287D | 749 | 131 | 117 | 763 | 85.91 | 0.85 | 0.87 | 0.72 | 0.92 |
| 289D | 748 | 132 | 119 | 761 | 85.74 | 0.85 | 0.86 | 0.71 | 0.92 |
| 291D | 752 | 128 | 117 | 763 | 86.08 | 0.85 | 0.87 | 0.72 | 0.92 |
| 293D | 751 | 129 | 120 | 760 | 85.85 | 0.85 | 0.86 | 0.72 | 0.93 |
| 295D | 752 | 128 | 113 | 767 | 86.31 | 0.85 | 0.87 | 0.73 | 0.93 |
| 297D | 751 | 129 | 115 | 765 | 86.14 | 0.85 | 0.87 | 0.72 | 0.93 |
| 299D | 750 | 130 | 116 | 764 | 86.02 | 0.85 | 0.87 | 0.72 | 0.93 |
| 301D | 753 | 127 | 113 | 767 | 86.36 | 0.86 | 0.87 | 0.73 | 0.93 |
| 303D | 754 | 126 | 115 | 765 | 86.31 | 0.86 | 0.87 | 0.73 | 0.93 |
| 305D | 751 | 129 | 116 | 764 | 86.08 | 0.85 | 0.87 | 0.72 | 0.92 |
| 307D | 751 | 129 | 116 | 764 | 86.08 | 0.85 | 0.87 | 0.72 | 0.92 |
| 309D | 751 | 129 | 117 | 763 | 86.02 | 0.85 | 0.87 | 0.72 | 0.92 |
| 311D | 751 | 129 | 116 | 764 | 86.08 | 0.85 | 0.87 | 0.72 | 0.92 |
| 313D | 751 | 129 | 116 | 764 | 86.08 | 0.85 | 0.87 | 0.72 | 0.92 |
| 315D | 751 | 129 | 116 | 764 | 86.08 | 0.85 | 0.87 | 0.72 | 0.92 |
| 317D | 751 | 129 | 117 | 763 | 86.02 | 0.85 | 0.87 | 0.72 | 0.92 |
| 319D | 748 | 132 | 118 | 762 | 85.80 | 0.85 | 0.87 | 0.72 | 0.92 |
| 321D | 748 | 132 | 112 | 768 | 86.14 | 0.85 | 0.87 | 0.72 | 0.92 |
| 323D | 749 | 131 | 112 | 768 | 86.19 | 0.85 | 0.87 | 0.72 | 0.92 |
| 325D | 749 | 131 | 111 | 769 | 86.25 | 0.85 | 0.87 | 0.73 | 0.92 |
| 327D | 748 | 132 | 114 | 766 | 86.02 | 0.85 | 0.87 | 0.72 | 0.92 |
| 329D | 747 | 133 | 114 | 766 | 85.97 | 0.85 | 0.87 | 0.72 | 0.92 |
| 331D | 748 | 132 | 116 | 764 | 85.91 | 0.85 | 0.87 | 0.72 | 0.92 |
| 333D | 747 | 133 | 118 | 762 | 85.74 | 0.85 | 0.87 | 0.71 | 0.92 |
| 335D | 748 | 132 | 117 | 763 | 85.85 | 0.85 | 0.87 | 0.72 | 0.92 |
| 337D | 748 | 132 | 117 | 763 | 85.85 | 0.85 | 0.87 | 0.72 | 0.92 |
| 339D | 748 | 132 | 117 | 763 | 85.85 | 0.85 | 0.87 | 0.72 | 0.92 |
| 341D | 748 | 132 | 119 | 761 | 85.74 | 0.85 | 0.86 | 0.71 | 0.92 |
| 343D | 748 | 132 | 116 | 764 | 85.91 | 0.85 | 0.87 | 0.72 | 0.92 |
| 345D | 748 | 132 | 116 | 764 | 85.91 | 0.85 | 0.87 | 0.72 | 0.92 |
| 347D | 748 | 132 | 116 | 764 | 85.91 | 0.85 | 0.87 | 0.72 | 0.92 |
| 349D | 748 | 132 | 119 | 761 | 85.74 | 0.85 | 0.86 | 0.71 | 0.92 |
| 351D | 748 | 132 | 118 | 762 | 85.80 | 0.85 | 0.87 | 0.72 | 0.92 |
| 353D | 748 | 132 | 118 | 762 | 85.80 | 0.85 | 0.87 | 0.72 | 0.92 |
| 355D | 748 | 132 | 119 | 761 | 85.74 | 0.85 | 0.86 | 0.71 | 0.92 |
| 357D | 746 | 134 | 116 | 764 | 85.80 | 0.85 | 0.87 | 0.72 | 0.92 |
| 359D | 748 | 132 | 118 | 762 | 85.80 | 0.85 | 0.87 | 0.72 | 0.92 |
| 361D | 747 | 133 | 119 | 761 | 85.68 | 0.85 | 0.86 | 0.71 | 0.92 |
| 363D | 748 | 132 | 119 | 761 | 85.74 | 0.85 | 0.86 | 0.71 | 0.92 |
| 365D | 749 | 131 | 117 | 763 | 85.91 | 0.85 | 0.87 | 0.72 | 0.92 |
| 367D | 750 | 130 | 115 | 765 | 86.08 | 0.85 | 0.87 | 0.72 | 0.92 |
| 369D | 748 | 132 | 116 | 764 | 85.91 | 0.85 | 0.87 | 0.72 | 0.92 |
| 371D | 749 | 131 | 117 | 763 | 85.91 | 0.85 | 0.87 | 0.72 | 0.92 |
| 373D | 749 | 131 | 118 | 762 | 85.85 | 0.85 | 0.87 | 0.72 | 0.92 |
| 375D | 749 | 131 | 118 | 762 | 85.85 | 0.85 | 0.87 | 0.72 | 0.92 |
| 377D | 749 | 131 | 117 | 763 | 85.91 | 0.85 | 0.87 | 0.72 | 0.92 |
| 379D | 749 | 131 | 116 | 764 | 85.97 | 0.85 | 0.87 | 0.72 | 0.92 |
| 381D | 748 | 132 | 116 | 764 | 85.91 | 0.85 | 0.87 | 0.72 | 0.92 |
| 383D | 749 | 131 | 120 | 760 | 85.74 | 0.85 | 0.86 | 0.71 | 0.92 |
| 385D | 749 | 131 | 120 | 760 | 85.74 | 0.85 | 0.86 | 0.71 | 0.92 |
| 387D | 749 | 131 | 120 | 760 | 85.74 | 0.85 | 0.86 | 0.71 | 0.92 |
| 389D | 749 | 131 | 121 | 759 | 85.68 | 0.85 | 0.86 | 0.71 | 0.92 |
| 391D | 748 | 132 | 120 | 760 | 85.68 | 0.85 | 0.86 | 0.71 | 0.92 |
| 393D | 748 | 132 | 120 | 760 | 85.68 | 0.85 | 0.86 | 0.71 | 0.92 |
| 395D | 748 | 132 | 120 | 760 | 85.68 | 0.85 | 0.86 | 0.71 | 0.92 |
| 397D | 749 | 131 | 118 | 762 | 85.85 | 0.85 | 0.87 | 0.72 | 0.92 |
| 399D | 749 | 131 | 118 | 762 | 85.85 | 0.85 | 0.87 | 0.72 | 0.92 |
| 401D | 747 | 133 | 117 | 763 | 85.80 | 0.85 | 0.87 | 0.72 | 0.92 |
| 403D | 748 | 132 | 117 | 763 | 85.85 | 0.85 | 0.87 | 0.72 | 0.92 |
| 405D | 748 | 132 | 117 | 763 | 85.85 | 0.85 | 0.87 | 0.72 | 0.92 |
| 407D | 747 | 133 | 118 | 762 | 85.74 | 0.85 | 0.87 | 0.71 | 0.92 |
| 409D | 747 | 133 | 117 | 763 | 85.80 | 0.85 | 0.87 | 0.72 | 0.92 |
| 411D | 747 | 133 | 118 | 762 | 85.74 | 0.85 | 0.87 | 0.71 | 0.92 |
| 413D | 747 | 133 | 119 | 761 | 85.68 | 0.85 | 0.86 | 0.71 | 0.92 |
| 415D | 746 | 134 | 119 | 761 | 85.63 | 0.85 | 0.86 | 0.71 | 0.92 |
| 417D | 747 | 133 | 119 | 761 | 85.68 | 0.85 | 0.86 | 0.71 | 0.92 |
| 419D | 746 | 134 | 118 | 762 | 85.68 | 0.85 | 0.87 | 0.71 | 0.92 |
| 421D | 748 | 132 | 118 | 762 | 85.80 | 0.85 | 0.87 | 0.72 | 0.92 |
| 423D | 748 | 132 | 118 | 762 | 85.80 | 0.85 | 0.87 | 0.72 | 0.92 |
| 425D | 748 | 132 | 118 | 762 | 85.80 | 0.85 | 0.87 | 0.72 | 0.92 |
| 427D | 748 | 132 | 119 | 761 | 85.74 | 0.85 | 0.86 | 0.71 | 0.92 |
| 429D | 748 | 132 | 117 | 763 | 85.85 | 0.85 | 0.87 | 0.72 | 0.92 |
| 431D | 746 | 134 | 120 | 760 | 85.57 | 0.85 | 0.86 | 0.71 | 0.92 |
| 433D | 745 | 135 | 117 | 763 | 85.68 | 0.85 | 0.87 | 0.71 | 0.92 |
| 435D | 745 | 135 | 118 | 762 | 85.63 | 0.85 | 0.87 | 0.71 | 0.92 |
| 437D | 750 | 130 | 127 | 753 | 85.40 | 0.85 | 0.86 | 0.71 | 0.92 |
| 439D | 749 | 131 | 126 | 754 | 85.40 | 0.85 | 0.86 | 0.71 | 0.92 |
| 441D | 749 | 131 | 126 | 754 | 85.40 | 0.85 | 0.86 | 0.71 | 0.92 |
| 443D | 749 | 131 | 126 | 754 | 85.40 | 0.85 | 0.86 | 0.71 | 0.92 |
| 445D | 748 | 132 | 126 | 754 | 85.34 | 0.85 | 0.86 | 0.71 | 0.92 |
| 447D | 744 | 136 | 122 | 758 | 85.34 | 0.85 | 0.86 | 0.71 | 0.92 |
| 449D | 745 | 135 | 122 | 758 | 85.40 | 0.85 | 0.86 | 0.71 | 0.92 |
| 451D | 752 | 128 | 128 | 752 | 85.45 | 0.85 | 0.85 | 0.71 | 0.92 |
| 453D | 752 | 128 | 128 | 752 | 85.45 | 0.85 | 0.85 | 0.71 | 0.92 |
| 455D | 753 | 127 | 124 | 756 | 85.74 | 0.86 | 0.86 | 0.71 | 0.92 |
| 457D | 754 | 126 | 124 | 756 | 85.80 | 0.86 | 0.86 | 0.72 | 0.92 |
| 459D | 751 | 129 | 123 | 757 | 85.68 | 0.85 | 0.86 | 0.71 | 0.92 |
| 461D | 753 | 127 | 125 | 755 | 85.68 | 0.86 | 0.86 | 0.71 | 0.92 |
| 463D | 751 | 129 | 125 | 755 | 85.57 | 0.85 | 0.86 | 0.71 | 0.92 |
| 465D | 752 | 128 | 125 | 755 | 85.63 | 0.85 | 0.86 | 0.71 | 0.92 |
| 467D | 752 | 128 | 123 | 757 | 85.74 | 0.85 | 0.86 | 0.71 | 0.92 |
| 469D | 750 | 130 | 124 | 756 | 85.57 | 0.85 | 0.86 | 0.71 | 0.92 |
| 471D | 750 | 130 | 126 | 754 | 85.45 | 0.85 | 0.86 | 0.71 | 0.92 |
| 473D | 750 | 130 | 126 | 754 | 85.45 | 0.85 | 0.86 | 0.71 | 0.92 |
| 475D | 752 | 128 | 125 | 755 | 85.63 | 0.85 | 0.86 | 0.71 | 0.92 |
| 477D | 752 | 128 | 124 | 756 | 85.68 | 0.85 | 0.86 | 0.71 | 0.92 |
| 479D | 752 | 128 | 124 | 756 | 85.68 | 0.85 | 0.86 | 0.71 | 0.92 |
| 481D | 753 | 127 | 128 | 752 | 85.51 | 0.86 | 0.85 | 0.71 | 0.92 |
| 483D | 754 | 126 | 131 | 749 | 85.40 | 0.86 | 0.85 | 0.71 | 0.92 |
| 485D | 753 | 127 | 130 | 750 | 85.40 | 0.86 | 0.85 | 0.71 | 0.92 |
| 487D | 749 | 131 | 129 | 751 | 85.23 | 0.85 | 0.85 | 0.70 | 0.92 |
| 489D | 750 | 130 | 128 | 752 | 85.34 | 0.85 | 0.85 | 0.71 | 0.92 |
| 491D | 746 | 134 | 127 | 753 | 85.17 | 0.85 | 0.86 | 0.70 | 0.92 |
| 493D | 750 | 130 | 133 | 747 | 85.06 | 0.85 | 0.85 | 0.70 | 0.92 |
| 495D | 751 | 129 | 133 | 747 | 85.11 | 0.85 | 0.85 | 0.70 | 0.92 |
| 497D | 743 | 137 | 126 | 754 | 85.06 | 0.84 | 0.86 | 0.70 | 0.92 |
| 499D | 750 | 130 | 133 | 747 | 85.06 | 0.85 | 0.85 | 0.70 | 0.92 |
| 501D | 744 | 136 | 126 | 754 | 85.11 | 0.85 | 0.86 | 0.70 | 0.92 |
| 503D | 752 | 128 | 134 | 746 | 85.11 | 0.85 | 0.85 | 0.70 | 0.92 |
| 505D | 750 | 130 | 134 | 746 | 85.00 | 0.85 | 0.85 | 0.70 | 0.92 |
| 507D | 749 | 131 | 132 | 748 | 85.06 | 0.85 | 0.85 | 0.70 | 0.92 |
| 509D | 743 | 137 | 128 | 752 | 84.94 | 0.84 | 0.85 | 0.70 | 0.92 |

**Table S3.** Comparison of different kernel functions of SVM

| Kernel | Sn(%) | Sp(%) | ACC(%) | MCC | AUC |
| --- | --- | --- | --- | --- | --- |
| RBF | 86.93 | 87.95 | 87.44 | 0.75 | 0.929 |
| Linear | 83.86 | 85.80 | 84.83 | 0.70 | 0.915 |
| Polynomial | 85.45 | 87.16 | 86.31 | 0.73 | 0.928 |
| Sigmoid | 84.55 | 85.45 | 85.00 | 0.70 | 0.915 |

**Table S4.** Comparison with other classifiers

| Classifier | Sn(%) | Sp(%) | ACC(%) | MCC | AUC |
| --- | --- | --- | --- | --- | --- |
| SVM | 86.93 | 87.95 | 87.44 | 0.75 | 0.929 |
| GBDT | 85.23 | 86.14 | 85.68 | 71.37 | 0.919 |
| RF | 80.11 | 80.57 | 80.34 | 60.68 | 0.882 |
| NB | 83.30 | 85.23 | 84.26 | 68.54 | 0.914 |
| LR | 85.23 | 85.34 | 85.28 | 70.57 | 0.918 |
| KNN | 86.14 | 80.68 | 83.41 | 66.92 | 0.901 |
